# Comparing the impact of “The Daily Mile™” vs. a modified version on Irish primary school children's engagement and enjoyment in structured physical activity

**DOI:** 10.3389/fspor.2025.1550028

**Published:** 2025-03-24

**Authors:** Luke Hanna, Con Burns, Cian O’Neill, Lisa E. Bolger, Edward Coughlan

**Affiliations:** Department of Sport, Leisure and Childhood Studies, Munster Technological University, Cork, Ireland

**Keywords:** The Daily Mile, The Daily Move, physical activity, health, enjoyment, engagement, primary school, children

## Abstract

**Introduction:**

The repetitive nature of The Daily Mile may affect long-term engagement. This study compared the impact of The Daily Mile with a modified version on engagement and enjoyment of structured physical activity.

**Methods:**

A ∼7-year-old and ∼11-year old cohort from six primary schools participated in this study, which primarily evaluated pre- to post-assessment changes within each group. Two schools were assigned to The Daily Mile (*n* = 102 children; *M* = 8.81 years, *SD* = 1.97), two schools to the modified version (*n* = 87; *M* = 9.16 years, *SD* = 2.76), and two schools served as controls (*n* = 79; *M* = 10.05 years, *SD* = 1.9) for 10-weeks. Health assessments conducted included cardiorespiratory fitness (550 m Run), fundamental movement skills (Test of Gross Motor Development-2), and health-related quality of life (KIDSCREEN-27). Accelerometers measured school-based physical activity, while enjoyment was assessed using a modified PACES questionnaire. Post-intervention focus groups (*n* = 10) included teachers and children.

**Results:**

Both the Daily Mile (∼7-year-old: *p* < 0.001; ∼11-year-old: *p* = 0.004) and its modified version (∼7-year-old: *p* < 0.001; ∼11-year-old: *p* < 0.001) had a significant impact on cardiorespiratory fitness. Participation in the modified version led to significant improvements in fundamental movement skills (∼7-year-old: *p* = 0.034; ∼11-year-old: *p* < 0.001), unlike participation in The Daily Mile (∼7-year-old: *p* = 1.000; ∼11-year-old: *p* = 0.807). A significant positive effect on health-related quality of life was attributed to participation in the modified version (*p* = 0.036), but not to The Daily Mile (*p* = 0.205). Enjoyment scores were significantly higher for the modified version (4.61 vs. 4.43; *p* = 0.024).

**Discussion:**

Participation in The Daily Mile is associated with improved health outcomes. Nevertheless, modifying the initiative to include greater variety has the potential to offer broader health benefits, longer engagement, and increased enjoyment. Future research should explore the long-term implementation of this modified version in schools.

## Introduction

Irish children and adolescents, aged between 5 and 17 years old, are recommended to engage in at least 60 min of moderate-to-vigorous physical activity (MVPA) per day each week (1). It has been reported that 23% of Irish primary school children meet these recommended PA guidelines (2). Notably, however, these findings are based on outdated guidelines recommending that Irish children engage in at least 60 min of MVPA daily (2). The updated PA guidelines, which state children may accumulate an average of at least 60 min of MVPA daily across the week, may be more attainable (1). PA has extensive positive effects on various aspects of children's health (3). These effects include improved body composition, cardiovascular health (4), physical fitness and motor competence (5) and lower risk of obesity (6).

Irish primary school children spend a considerable portion of their waking hours at school, attending for nearly 6 h every weekday (7), while also being advised to avoid engaging in sedentary behaviour for prolonged periods (1, 8). This indicates that school is an appropriate setting to enhance children's PA levels. School-based PA interventions have reported positive effects for lowering the risk and prevalence of obesity among children (9). Furthermore, Kelso et al. (10) reported that school-based PA interventions can improve children's PA behaviour by positively affecting motivational factors such as enjoyment, perceived autonomy, identified regulation, and intrinsic motivation. School-based PA interventions have been previously shown to positively impact children's PA behaviour and physical fitness (11), fundamental movement skill (FMS) proficiency (12), mental health (13), cognitive function, and psychosocial wellbeing (14).

The Daily Mile (TDM) is a school-based PA initiative that was developed in a Scottish primary school in 2012 to address children's low fitness levels (15). It has since been adopted and implemented by over 21,000 schools across 98 countries, with over 1,350 Irish primary schools now registered as TDM participants (16). According to the implementation principles of TDM (16), the 15 min initiative should be enjoyable and non-competitive, requiring no additional time or equipment to set up. Children are encouraged to run or jog at their own pace on a mud-free surface for the entire 15 min, taking short walking breaks to catch their breath if needed (16). Extensive research on TDM has been published previously, reporting a positive association between the initiative and markers of children's physical health including their cardiorespiratory fitness (CRF) and PA behaviour (17, 18). According to Morris et al. (19) and Hatch et al. (20), participating in a TDM session does not have an immediate significant effect on children's executive functions. Conversely, participating in TDM for a period of five weeks has been shown to significantly improve children's response time on the complex level of the Stroop test, which is designed to measure inhibitory control (21). These findings suggest that positive developments in children's cognitive processes may only be observed after long-term participation in TDM. Furthermore, TDM may positively impact components that affect children's mental health, particularly for those experiencing poor mental health before participating (22). In support, TDM has been recognised for positively impacting children's psychological health metrics such as wellbeing, self-esteem, stress relief, and academic performance (23). Moreover, TDM has been reported to positively affect children's social health and promotes the development of a strong social bond between children and their teachers (24, 25). Although no research has directly assessed the impact of TDM on children's health-related quality of life (HRQoL), these existing studies highlight positive effects on psychological and social health, suggesting a positive potential relationship.

The implementation of TDM and its perceived health benefits within Irish primary schools has been well-received by teachers, principals, and children alike (25). Supporting this, research by Breslin et al. (26) indicates that over half (54.7%) of the primary schools in Northern Ireland regularly implement TDM. Similarly, a comparable proportion of schools in London (53%) and Leicester (59.5%), England, have been reported as implementers of TDM (27, 28). However, research suggests that the core TDM implementation principles are not always adhered to over time (26, 28). Similarly, Herlitz et al. (29) reported that the original format of each school-based intervention assessed (*n* = 18) in their research was not fully sustained over time. These findings infer that bespoke modifications to initiatives such as TDM are required to ensure their sustainability and to align with the unique characteristics of participating schools and children. Additionally, some children desire a greater variety and choice of PA options when participating in TDM to maximise their engagement and enjoyment with the initiative (20, 25). Moreover, research suggests that teachers adapt TDM with games and competitive elements to better meet their class's needs and preferences, ensuring sustained engagement and motivation over time (25, 27, 30). Similarly, Project Spraoi, a multi-faceted primary school-based PA and nutrition intervention, was widely regarded by teachers as having a positive impact on children's fitness levels and eating behaviours. However, barriers such as inclement weather, curriculum pressures, time restrictions, and limited resource access prevented teachers from consistently delivering 20 min of MVPA daily, as outlined in the intervention's implementation principles (31). Furthermore, the delivery of PA sessions was not sustained by teachers once the specialist external coordinator was no longer available to lead or oversee them (32). These findings suggest that primary school-based PA initiatives must be simple and manageable for teachers to implement while also adaptable enough to overcome common barriers and sustain engagement over time.

Scannell and Murphy (33) adopted a qualitative approach to examine how modifying TDM affects children's engagement and enjoyment. The findings from this study indicate that incorporating additional activities beyond running or jogging may enhance both engagement and enjoyment (33). However, no research has assessed or compared the impact of TDM on children's health-related metrics and enjoyment with a modified version of the initiative that offers greater variety and choice of PA options during the 15 min session. To address this gap in research, The Daily Move (TDMo) was developed as an evolution of the traditional version of The Daily Mile (TDMi). This development was guided by the Self-Determination Theory framework, which identifies the psychological needs that influence an individual's motivation to engage with an activity (34). Intrinsic motivation, widely recognised as a significant predictor of human behaviour, cannot occur without a feeling of autonomy in one's actions (34). Sebire et al. (35) supported this by reporting that autonomy among children (*n* = 462; 56.9% girls; *M* = 10.03 years, *SD* = 0.57) who do not regularly participate in school sports teams positively influenced their intrinsic motivation, which in turn was positively associated with MVPA. Comparably, autonomy, along with competence and relatedness, has been shown to significantly enhance children's autonomous motivation (*n* = 1,665, 830 girls; *M* *=* 12.43 years, *SD* = 1.87) and intention to engage in PA (36). Furthermore, the provision of new and original activities has been found to significantly influence children's autonomous motivation and intention for positive PA behaviour (36). Similarly, school-based interventions that offer children various PA options when participating can satisfy their need for autonomy and improve their MVPA engagement (37). TDMo requires teachers and children to collaborate in selecting a variety of activities that stimulate inclusive engagement and motivate participation from everyone in the class. Additionally, inclement weather, which often creates unsuitable and unsafe outdoor surfaces for exercise, has frequently been acknowledged as a barrier to the implementation of TDMi (23, 25, 38). Furthermore, a shortage of outdoor space can impact the delivery of TDMi (15, 27). In response, TDMo includes PA options for indoor delivery, within sports halls or classrooms, ensuring movement break opportunities are maintained during unfavourable outdoor conditions.

No research has been previously conducted that explores the impact of TDMi on children's FMS. Research should adopt a holistic approach when measuring and comparing the impact of TDMi with a modified version on markers of children's health, engagement, and enjoyment. Therefore, the objectives of this research were to accomplish the following: (1) evaluate and compare the impact of TDMi and TDMo on children's health-related metrics including PA behaviour, CRF, FMS, and health-related quality of life (HRQoL), (2) evaluate and compare the impact of participation in TDMi and TDMo on children's engagement and enjoyment levels, and (3) explore and compare the perceptions of children and teachers regarding the implementation of TDMi and TDMo.

## Materials and methods

### Study design

A quasi-experimental study was conducted using a between-subjects design. Convenience sampling was used to recruit six primary schools in Cork, Ireland, based on their willingness to participate and logistical feasibility. Each participating school's principal designated two class groups to participate from teachers who expressed an interest: one ∼7-year-old cohort (junior and senior infants, as well as first and second class) and one ∼11-year-old cohort (third, fourth, fifth, and sixth class). Two schools with previous experience of implementing The Daily Mile (TDMi), were assigned to participate in The Daily Move (TDMo) condition to account for prior exposure to TDMi. The remaining four schools, who had no previous experience of implementing TDMi, were randomly assigned to the two remaining conditions. Two of these schools participated in TDMi condition, while the other two schools served as the control group. This assignment strategy aimed to minimise potential confounding effects of prior engagement with TDMi when comparing conditions.

**Figure 1:**
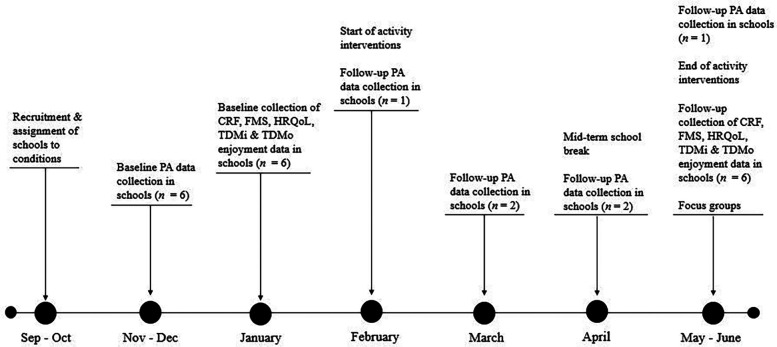
Research timeline.

Schools participated in their assigned conditions for 10 weeks, with a 2-week mid-term break at Easter while the school observed a national holiday. During the mid-term break, TDMi and TDMo participants received a PA report card with 17 dates and stickers to track and record any day they engaged in at least 60 min of PA. The primary researcher and research assistant implemented TDMi and TDMo initiatives with the classes assigned to these conditions, three days every week during school hours, alongside scheduled physical education (PE) classes. Teachers of these classes were encouraged to implement their assigned initiative on the remaining days of the week. The control schools were instructed not to implement either TDMi or TDMo with their participating class groups during the 10-week intervention. Figure 1 presents a visual flowchart illustrating the research timeline and highlights key data collection events.

### Participants

A total of 268 children (*n* = 132 females, 136 males) participated in this study. Baseline characteristics of the participants are displayed in Table 1. Kruskal Wallis tests revealed no significant difference in sex distribution between the ∼7-year-old groups (*p* = 0.746), or the ∼11-year-old groups (*p* *=* 0.642). Kruskal–Wallis tests found no significant differences in Body Mass Index (BMI) among the ∼7-year-old groups (*p* = 0.545), or the ∼11-year-old groups (*p* = 0.846). However, Kruskal–Wallis and Mann–Whitney U tests indicated significant age differences within the ∼7-year-old (*p* < 0.001) and ∼11-year-old (*p* < 0.001) groups.

**Table 1:** Baseline characteristics of study participants.

Participant characteristics	TDMi		TDMo		Control	
	∼7-year-old	∼11-year-old	∼7-year-old	∼11-year-old	∼7-year-old	∼11-year-old
Male (%)	28 (50)	21 (45.7)	24 (54.5)	23 (53.5)	22 (57.9)	18 (43.9)
Female (%)	28 (50)	25 (54.3)	20 (45.5)	20 (46.5)	16 (42.1)	23 (56.1)
Age (SD)	7.26 (0.46)	10.70 (1.34)	6.65 (1.37)	11.73 (0.64)**	8.17 (0.52)*	11.80 (0.49)**
BMI (SD)	16.46 (2.15)	18.28 (2.49)	16.39 (2.03)	18.33 (2.66)	16.81 (2.36)	18.77 (3.08)

* Significantly older than the ∼7-year-old TDMi and TDMo groups, *p* *<* 0.001.

** Significantly older than the ∼11-year-old TDMi group, *p* < 0.001.

The names of all consenting children and schools were coded to ensure participants' anonymity was maintained throughout and after the study. All data collected were gathered in the presence of the teacher or school staff member. Data were stored securely on a password-protected laptop and external hard drive, both accessible only to the primary researcher, to uphold confidentiality throughout and beyond the study. Informed consent was obtained from all participants and their parents, and ethical approval (MTU21023A) was secured from the host institute before data collection began. All children from participating schools in the ∼7-year-old and ∼11-year-old cohorts were eligible to participate, provided both the child and their parents gave informed consent prior to the commencement of baseline data collection. Children who were injured at either the baseline or follow-up assessment were not permitted to participate in the health measurements.

### The Daily Mile (TDMi)

Participants adhered to the ten core implementation principles of The Daily Mile Foundation (16), which involved walking or running at a self-regulated pace for 15 min during the initiative. Every TDMi session was implemented outside. It was not delivered on days when common implementation barriers, such as inclement weather, prevented children's safe engagement in outdoor PA; thereby, no indoor activities were conducted.

### The Daily Move (TDMo)

Participants adhered to the implementation principles that supported the development of TDMo during its delivery (Table 2). Participants engaged in a variety of different games and activities for 15 min which included relays, tag games, and ball games. During days where common implementation barriers, such as inclement weather, prevented children's safe engagement with PA outside, activities were conducted in the school's indoor hall or classroom. The activities delivered varied throughout the week to accommodate the perceived needs and preferences of each class group.

**Table 2:** The 8 core implementation principles of The Daily Move.

No.	Principle	Description
1	Daily delivery	Teachers are encouraged to deliver the initiative during every school day, especially on days with no scheduled Physical Education (PE) classes.
2	Outdoor is best	Outdoor activity should be prioritised. However, movement breaks should be conducted indoors in a hall or classroom during adverse weather conditions or when safe outdoor spaces are unavailable for children to engage in physical activity.
3	Inclusive	The participation of all children, including those with varying needs and abilities, is fundamental to the delivery of any game or activity. Games or activities should be adapted or modified as necessary, to ensure the full inclusion and participation of all children in the class.
4	Keep it simple	Games and activities should not be overcomplicated. It is important that all children understand the rules before starting to ensure they enjoy participating and have the opportunity to maximise their physical activity output.
5	15 min movement break	All activities and games should be completed within 15 min to minimise any negative impact on children's learning time in the classroom.
6	Variety and choice	To sustain children's motivation and engagement over time, it is essential to implement a variety of novel games and activities during each 15 min movement break.
7	Teamwork	Teachers, staff, and children are encouraged to work together towards one common goal: ensuring that all children feel included and enjoy participating in The Daily Move.
8	Be creative	Creativity in planning each 15 min movement break is strongly encouraged. Suggestions from children to modify or adapt existing games and activities to enhance their engagement and enjoyment should be welcomed and supported.

### Participant assessments

Participants' height and weight were measured to the nearest 0.1 cm and 0.1 kg, using a Leicester portable stadiometer and a Tanita WB100MZ portable electronic scale, before the start of the research conditions. Pre- and post-test assessments of CRF, FMS, and HRQoL were collected in January 2023 and May 2023. Participants' wore accelerometers for five days before the research conditions began, and again for five days during participation in their assigned research conditions. This allowed for an evaluation of how each condition impacted their PA behaviour at school. The ∼11-year-old TDMi and TDMo participants completed a post-participation questionnaire to assess their enjoyment of each initiative.

#### 550 m Run

A 550 m Run test was administered to measure children's CRF (39). This test required participants to complete five laps of a circuit with a circumference of 110 m as fast as possible. Four markers were equally spaced out around the circuit. Before each trial began, one participant stood at each marker. All children participating in that 550 m trial (i.e., 4 children) were instructed to move around the circuit at a pace they could sustain for five laps without stopping. A whistle was blown to signal the start of the test, and the time each child took to complete the 550 m distance was recorded in seconds. These times provided an indirect measure of CRF, with faster completion times reflecting higher levels of CRF.

#### The test of gross motor development-second edition (TGMD-2)

The TGMD-2 was used to measure children's FMS competence (40). TGMD-2 is comprised of two sets of sub-skills; locomotor and object-control. The six locomotor skills evaluated are the run, gallop, hop, leap, slide, and horizontal jump. The six object-control skills evaluated are the overhand throw, catch, underhand roll, kick, strike, and stationary dribble. On the days of testing, the indoor school hall of each participating class group was split into four sections with the following skills tested at each station: (1) run, gallop, and leap; (2) hop, slide, and jump; (3) catch, throw, and roll; (4) kick, strike, and dribble. The participants from each class were divided into four groups and each group rotated clockwise through the four stations. Participants completed three trials for each skill: one practice trial and two test trials. Each trial was recorded on a video camera to facilitate the review and scoring of participants' FMS performance. The videos were then uploaded to a laptop. If participants performed a component of the skill correctly, they were awarded a score of 1. A score of 0 was awarded for any skill component that was performed incorrectly. Scores from both trials were combined to determine a raw score for each skill. The raw skill scores within each subset were then added together to calculate a measure of participants' locomotor and object-control skills. An overall FMS index score was established by combining participants' locomotor and object-control scores. Inter- and intra-rater reliability were calculated off 10% of the sample using the equation [agreements/(agreements + disagreements) ×  100] (41). The primary researcher and a practitioner with extensive TGMD-2 experience calculated inter-rater reliability by video analysis of the recorded FMS trials. The inter- and intra-rater reliability scores exceeded the required agreement threshold of 85% (41), ranging from 86%–100%.

#### Physical activity and sedentary behaviour

Physical activity, sedentary behaviour, and children's mean daily step count during school time were objectively measured using GT3X ActiGraph accelerometers. Children wore the accelerometers for five consecutive school days, both before and during their assigned research conditions, allowing for an analysis of the impact that each condition had on these measures. The accelerometers were distributed to each participating class group at the start of the school week. The class teacher received a message on WhatsApp at the start of each day to remind children to put on their accelerometers. The accelerometer was attached to a belt which children wore around their waist on their left hip. Children handed their accelerometer back to their teacher at the end of each day. The accelerometer data was downloaded to ActiLife software by the primary researcher to facilitate the analysis of collected data. Inclusion criteria required children to wear the accelerometers for ≥3 school days and for at least 70% of the time during the school day (42). The threshold for inclusion in the analysis was a daily wear time of ≥4 h to ensure sufficient data was captured to accurately reflect school-day activity patterns. However, for the ∼7-year-old TDMi and TDMo class groups in their first two years of primary school, a reduced daily wear time of ≥3 h and 20 min was considered acceptable. This adjustment accounted for the one-hour shorter school day commonly observed for Irish children in their first two years of primary school (7). A 5 s epoch was used to capture the intermittent nature of children's PA behaviour (43). Non-wear time was defined at 20 min of consecutive zeroes, following established protocols (44). Sedentary time and MVPA were calculated using cut-points developed by Evenson et al. (45) and validated by Trost et al. (46), ensuring robust classification of activity intensities. Missing data were managed by excluding any days that did not meet the minimum wear-time criteria. Participants with insufficient valid days of wear time were excluded from the final analysis.

#### Health-related quality of life (HRQoL)

The impact of TDMi and TDMo on children's HRQoL was measured using the validated KIDSCREEN-27 questionnaire which encompasses various aspects of children's health-related wellbeing (47). The questionnaire was administered to the ∼11-year-old groups, pre- and post-participation in their assigned research conditions. The ∼7-year-old groups did not complete the questionnaire, as it has not been validated for children under 8 years old (47). KIDSCREEN-27 is comprised of the following five sections: (1) physical wellbeing; (2) psychological wellbeing; (3) autonomy and parental relations; (4) peers and support; (5) school environment. The number of questions within each section that participants answered using a 5-point Likert scale ranged between four to seven. The Likert-scale responses were valued from one to five. The scores for each question were added together to provide a HRQoL score for each participant, with higher scores indicating better health-related wellbeing (Supplementary material S1).

#### TDMi and TDMo enjoyment

A modified version of the validated Physical Activity Enjoyment Scale (PACES) questionnaire was administered to TDMi and TDMo participants at the end of the study (48). This modified questionnaire measured the level of enjoyment that children associated with participating in TDMi and TDMo (Supplementary material S2). The modified questionnaire retained the 16 statements from PACES but adapted the stem to “When I participate in The Daily Mile …” or “When I participate in The Daily Move …”. Participants rated their agreement with each statement using a 5-point Likert scale (i.e., “disagree a lot” to “agree a lot”). The Likert-scale responses to each statement were valued from one to five, and an index score for participants' enjoyment was calculated by averaging the scores of the 16 items. Mean modified PACES questionnaire scores were compared between TDMi and TDMo groups, with higher scores indicating greater enjoyment of the respective initiative. Additionally, six new statements, formatted similarly to the original 16 items (i.e., “When I participate in TDMi/TDMo …”), focused on the implementation and perceived impact of TDMi and TDMo. These additional responses did not influence the calculation of the mean enjoyment score for TDMi and TDMo. Responses from the ∼7-year-old groups were excluded from analysis because a considerable proportion of children completed the questionnaire incorrectly.

The participating children completed the questions as a group, one questionnaire at a time (i.e., KIDSCREEN-27 followed by the modified PACES questionnaire). The primary researcher was present during the administration to promptly address any queries.

#### Focus groups

These (*n* = 8) were held with a sample of children from the ∼7-year-old and ∼11-year-old groups that participated in TDMi and TDMo. Six children were randomly selected to participate in each focus group by their teacher. The questions were designed to further explore the implementation and impact of both initiatives on children's enjoyment and health-related metrics (Table 3). The child-centred focus groups were held in the school's facilities. Two focus groups were also held with teachers of the classes that participated on the online Zoom platform (San Jose, CA, USA). These focus groups were designed to evaluate the implementation, effectiveness, and sustainability of TDMi and TDMo. Three teachers participated in the TDMo-centred focus group and four teachers participated in the TDMi-centred focus group. Additional prompting questions were used by the primary researcher during the child and teacher-centred focus groups if the initial question did not elicit a clear and detailed response.

**Table 3:** Focus group question*s.*

No.	Child focus groups	Teacher focus groups
Q1.	What do you most enjoy about participating in TDMi/TDMo?	What are the main strengths of TDMi/TDMo?
Q2	How did participating in the TDMi/TDMo impact your health & wellbeing?	What are the main weaknesses of TDMi/TDMo?
Q3	Is there any part of TDMi/TDMo that you do not enjoy? If so, what do you least enjoy about participating in TDMi/TDMo?	Did you see any improvement to markers of children's health and wellbeing following participation in TDMi/TDMo?
Q4	Is there any part of TDMi/TDMo that you would change to make it more enjoyable to participate in? Please explain your answer.	How was children's behaviour in the classroom impacted following participation in TDMi/TDMo?
Q5	Is there any reason why your class would not participate in TDMi/TDMo on a given day? Please explain further any reason why your class might not participate in TDMi/TDMo.	If you had the opportunity, would you make any adjustments or changes to TDMi/TDMo to make it more enjoyable for participating children?
Q6	Would you like to see your school continue to implement TDMi/TDMo in the future? Please explain your answer.	What are the biggest threats to the sustained implementation of TDMi/TDMo with your class group?
Q7	N/A	Were there any elements of TDMi/TDMo that children perceived as boring or repetitive?
Q8	N/A	Do you plan on continuing to implement TDMi/TDMo with your class next year? Please explain your answer.

### Data analysis

The IBM Statistical Package for the Social Sciences (SPSS) version 29 (Chicago, IL, USA) was used for analysis after data coding and entry were completed. The data were cleaned to ensure accuracy across all variables before analysis. Descriptive statistics were calculated for baseline and follow-up measures across condition-age groups (i.e., ∼7-year-old TDMi, TDMo, and control groups; ∼11-year-old TDMi, TDMo, and control groups) for the 550 m Run, TGMD-2, and school-based PA and sedentary behaviour results. Additionally, descriptive statistics for baseline and follow-up KIDSCREEN-27 scores were calculated for the oldest condition-age groups (i.e., ∼11-year-old TDMi, TDMo, and control groups), and follow-up modified PACES scores were determined for the oldest condition-age intervention groups (i.e., ∼11-year-old TDMi and TDMo groups). To assess the normality of data distribution, Shapiro–Wilk goodness-of-fit tests were used prior to running statistical significance tests. A Linear Mixed Model was conducted to examine changes in measured health metrics from baseline to follow-up, with participants' school and sex controlled for as random effects in the model. Additionally, a Mann Whitney-U test was used to compare differences between groups for the not-normally distributed modified PACES data. The alpha level required for all tests of significance was set at *p* < 0.05. Cohen's d was manually calculated using the formula outlined by Brysbaert and Stevens (49), where effect size was determined by the difference between means divided by the average standard deviation of both variables. A threshold of ≥0.2 and <0.5 indicated a small effect, ≥0.5 and <0.8 a medium effect, and ≥ 0.8 a large effect (50). *a priori* power analysis indicated that a sample size of 18 participants was required for a Repeated Measures ANOVA test. Given the need to control for variability across schools and sex, a Linear Mixed Model was employed instead to appropriately model these as random effects. Furthermore, power analysis determined that 27 participants were needed for a Mann–Whitney *U* test to achieve 80% power at an alpha level of 0.05.

The primary researcher adhered to the six-step thematic analysis process outlined by Braun and Clarke (51) to interpret and analyse the qualitative data collected from the focus groups. This analysis was facilitated using NVivo software version 12 (Greenwood Village, CO, USA). Otter.ai software (Mountain View, CA, USA) was used to transcribe the dialogue from the focus group recordings. The transcription was then manually edited by the researcher to include syntax in Microsoft Word. During this initial phase of familiarisation with the data, the researcher noted common discussion points across the focus groups, which facilitated the creation of the coding framework on NVivo. Relevant aspects of the focus group data were coded and then organised under broader themes and sub-themes. These themes were reviewed to certify that the coded data accurately captured the essence and characteristics of each theme.

## Results

The results section examines the impact of TDMi, TDMo, and control conditions on the measured health metrics in both the ∼7-year-old and ∼11-year-old groups. The focus group data, which analyses the health impacts, enjoyment, implementation, and sustainability of TDMi and TDMo, is also presented. The implementation rate of TDMi ranged from 85%–91% across participating groups, while the rate for TDMo was slightly higher, ranging from 94%–98%.

### 550m Run

The mean results for participants who completed both baseline and follow-up 550 m run tests are presented in Table 4. One of the two participating control schools did not complete the baseline test before the research conditions began, and therefore, were omitted from the analysis. After controlling for children's school as a random effect, statistically significant improvements in mean 550 m Run performance from baseline to follow-up were observed in the ∼7-year-old TDMi (*F* = 12.106, *p* < 0.001) and TDMo groups (*F* = 22.911, *p* < 0.001), as well as in the ∼11-year-old TDMi (*F* = 8.700, *p* = 0.004) and TDMo groups (*F* = 15.625, *p* < 0.001). Conversely, improvements in the ∼7-year-old control (*F* = 1.111, *p* = 0.299) and ∼11-year-old control (*F* = 0.697, *p* = 0.409) groups were not statistically significant. Following adjustments for both children's sex and school as random effects, significant improvements remained in the ∼7-year-old TDMi (*F* = 13.003, *p* < 0.001), ∼7-year-old TDMo (*F* = 25.056, *p* < 0.001), ∼11-year-old TDMi (*F* = 9.085, *p* = 0.004), and ∼11-year-old TDMo groups (*F* = 17.368, *p* < 0.001), while the changes in the ∼7-year-old control (*F* = 1.322, *p* = 0.258) and ∼11-year old control (*F* = 0.642, *p* = 0.429) groups remained non-significant.

**Table 4:** Mean baseline and follow-up 550 m Run times in seconds.

Condition-age group	*N*	Baseline 550 m (SD)	Follow-up 550 m (SD)
∼7-year-old TDMi	49	219.08 (31.29)	197.02 (32.99)^b^
∼7-year-old TDMo	39	220.33 (29.53)	194.03 (26.85)^c^
∼7-year-old Control	20	187.10 (28.08)	178.50 (23.31)^a^
∼11-year-old TDMi	37	179.41 (19.02)	167.81 (17.51)^b^
∼11-year-old TDMo	40	172.15 (15.76)	159.43 (13.18)^c^
∼11-year-old Control	20	159.30 (11.07)	156.40 (10.90)^a^

^a^ Small effect size from baseline.

^b^ Medium effect size from baseline.

^c^ Large effect size from baseline.

### TGMD-2

Mean FMS results for participants who completed both baseline and follow-up tests are presented in Table 5. Following adjustments for school effects, significant improvements in mean FMS performance were observed in the ∼7-year old TDMo group (*F* = 4.686, *p* = 0.034) and the ∼11-year-old TDMo group (*F* = 13.748, *p* < 0.001). However, no significant changes were detected from baseline to follow-up in the ∼7-year-old TDMi (*F* = 0.000, *p* = 1.000) or control (*F* = 0.000, *p* = 0.986) groups, nor in the ∼11-TDMi (*F* = 0.060, *p* = 0.807) or ∼11-control (*F* = 1.903, *p* = 0.172) groups. After further adjustments for school and sex, significant differences between baseline and follow-up remained in the ∼7-year old TDMo (*F* = 4.686, *p* = 0.034) and in the ∼11-year-old TDMo (*F* = 14.018, *p* < 0.001) groups. In contrast, no significant changes were observed in the ∼7-year-old TDMi (*F* = 0.000, *p* = 1.000), ∼7-year-old control (*F* = 0.000, *p* = 0.986), ∼11-year-old TDMi (*F* = 0.060, *p* = 0.807), or ∼11-year-old control (*F* = 1.903, *p* = 0.172) groups.

**Table 5:** Mean baseline and follow-up TGMD-2 results.

Condition-age group	*N*	Baseline locomotor (SD)	Follow-up locomotor (SD)	Baseline object-control (SD)	Follow-up object-control (SD)	Baseline FMS total (SD)	Follow-up FMS total (SD)
∼7-year old TDMi	43	32.28 (3.10)	31.63 (3.39)^d^	27.37 (5.16)	28.02 (4.93)	59.65 (6.38)	59.65 (6.80)
∼7-year old TDMo	35^a^	29.66 (4.67)	32.00 (4.88)^d^	25.36 (8.23)	26.86 (6.33)^d^	54.94 (11.85)	58.77 (9.53)^d^
∼7-year old Control	33	29.61 (4.37)	30.24 (4.58)	29.79 (4.79)	29.18 (4.32)	59.39 (6.86)	59.42 (6.84)
∼11-year old TDMi	34^b^	34.88 (3.12)	33.88 (2.65)^d^	30.17 (5.00)	31.19 (5.03)^d^	64.68 (6.21)	65.03 (5.75)
∼11-year old TDMo	39	33.49 (3.28)	35.87 (3.90)^e^	33.49 (3.78)	35.10 (3.29)^d^	66.97 (5.13)	70.97 (4.43)^f^
∼11-year old Control	37^c^	31.66 (4.00)	33.84 (2.92)^e^	33.11 (3.91)	32.70 (3.37)	64.73 (6.50)	66.54 (4.99)^d^

^a^ 36 children in the ∼7-year old TDMo group completed both object-control assessments.

^b^ 36 children in the ∼11-year old TDMi group completed both object-control assessments.

^c^ 38 children in the ∼11-year old control group completed both locomotor assessments.

^d^ Small effect size from baseline.

^e^ Medium effect size from baseline.

^f^ Large effect size from baseline.

After adjusting for school as a random effect, significant improvements in mean locomotor performance from baseline to follow up were observed in the ∼7-year-old TDMo (*F* *=* 5.273, *p* = 0.025), ∼11-year-old TDMo (*F* *=* 8.529, *p* = 0.005), and ∼11-year-old control (*F* *=* 7.866, *p* = 0.006) groups. Conversely, no significant changes over time were exhibited among the ∼7-year-old TDMi (*F* *=* 0.932, *p* = 0.337), ∼7-year-old control (*F* *=* 0.345, *p* = 0.559), or ∼11-year-old TDMi (*F* *=* 2.029, *p* = 0.159) groups. Following further adjustments for both school and sex, significant improvements in locomotor performance were still evident in the ∼7-year-old TDMo (*F* *=* 5.273, *p* = 0.025), ∼11-year-old TDMo (*F* *=* 8.615, *p* = 0.004), and ∼11-year-old control (*F* *=* 8.499, *p* = 0.005) groups. Moreover, observed changes remained non-significant in the ∼7-year-old TDMi (*F* *=* 0.937, *p* = 0.336), ∼7-year-old control (*F* *=* 0.348, *p* = 0.557), and ∼11-year-old TDMi (*F* *=* 2.007, *p* = 0.161) groups.

Following adjustments for school effects, significant improvements in object-control performance from baseline to follow-up were observed in the ∼11-year-old TDMo group (*F* *=* 4.108, *p* = 0.046). No significant changes were found in the ∼7-year TDMi (*F* *=* 0.358, *p* = 0.551), TDMo (*F* *=* 1.742, *p* = 0.191), or control (*F* *=* 0.293, *p* = 0.590) groups, nor in the ∼11-year old TDMi (*F* *=* 0.816, *p* = 0.369) or control (*F* *=* 0.229, *p* = 0.633) groups. Furthermore, after adjusting for both school and sex, the ∼11-year-old TDMo group maintained a significant improvement (*F* *=* 4.108, *p* = 0.046), while all other groups' changes remained non-significant (∼7-year TDMi: *F* *=* 0.453, *p* = 0.503; ∼7-year-old TDMo: *F* *=* 1.742, *p* = 0.189; ∼7-year-old control: *F* *=* 0.308, *p* = 0.581; ∼11-year old TDMi: *F* *=* 0.816, *p* = 0.369; ∼11-year-old control: *F* *=* 0.231, *p* = 0.633).

### PA and sedentary behaviour

Results for the mean percentage of time spent in MVPA, sedentary behaviour, and the mean daily step count for each condition-age group are presented in Table 6. After controlling for school effects, significant improvements in the mean school-based percentage of time spent in MVPA were found in the ∼7-year-old TDMi (*F* = 5.001, *p* = 0.028), ∼7-year-old TDMo (*F* = 31.037, *p* < 0.001), ∼11-year-old TDMi (*F* = 7.312, *p* = 0.009), and ∼11-year-old TDMo (*F* = 9.423, *p* = 0.003) groups. The ∼7-year-old control (*F* = 0.018, *p* = 0.894) or ∼11-year-old control (*F* = 3.520, *p* = 0.065) groups did not experience a significant change from baseline to follow up. Further adjustments for the effects of school and gender confirmed these findings, with significant differences between baseline and follow-up remaining in the ∼7-year-old TDMi (*F* = 5.822, *p* = 0.018), ∼7-year-old TDMo (*F* = 36.716, *p* < 0.001), ∼11-year-old TDMi (*F* = 8.556, *p* = 0.005), and ∼11-year-old TDMo (*F* = 10.771, *p* = 0.002) groups. Additionally, a significant increase was observed in the ∼11-year-old control group (*F* = 5.218, *p* = 0.026), while the ∼7-year-old control group showed no significant difference between timepoints (*F* = 0.023, *p* = 0.881).

**Table 6:** Mean baseline and follow-up weekly school-based MVPA and sedentary percentages, and mean daily step count.

Condition-age group	*N*	Baseline mean MVPA % (SD)	Follow-up mean MVPA % (SD)	Baseline mean sedentary % (SD)	Follow-up mean sedentary % (SD)	Baseline mean step count (SD)	Follow-up mean step count (SD)
∼7-year-old TDMi	48	7.69 (2.24)	8.70 (2.21)^a^	65.93 (7.07)	64.67 (6.40)	3,940 (883)	4,766 (671)^c^
∼7-year-old TDMo	42	5.37 (1.84)	7.90 (2.34)^c^	71.05 (4.68)	67.44 (5.30)^b^	3,093 (809)	3,992 (1,029)^c^
∼7-year-old Control	34	8.87 (3.03)	8.95 (2.97)	67.33 (5.94)	67.80 (6.06)	4,293 (908)	4,838 (1,411)^a^
∼11-year-old TDMi	31	6.74 (2.48)	8.47 (3.63)^b^	70.06 (5.15)	68.65 (5.55)^a^	3,896 (1,227)	4,897 (1,471)^b^
∼11-year-old TDMo	35	6.28 (3.69)	8.30 (3.54)^b^	72.61 (8.29)	69.10 (7.27)^a^	3,715 (1,694)	4,385 (1,317)^a^
∼11-year-old Control	35	8.45 (2.99)	9.57 (2.80)^a^	67.61 (7.49)	67.94 (5.17)	4,392 (1,352)	5,109 (1,359)^b^

^a^ Small effect size from baseline.

^b^ Medium effect size from baseline.

^c^ Large effect size from baseline.

Following adjustments for school effects, significant reductions in the mean percentage of time spent in school-based sedentary activity were observed in the ∼7-year-old TDMo (*F* = 11.293, *p* = 0.001) and ∼11-year-old TDMo (*F* = 7.945, *p* = 0.006) groups. No significant changes were found in the ∼7-year-old TDMi (*F* = 1.030, *p* = 0.313), ∼7-year-old control (*F* = 0.131, *p* = 0.718), ∼11-year-old TDMi (*F* = 1.333, *p* = 0.253), or ∼11-year-old control (*F* = 0.063, *p* = 0.803) groups. After further adjusting for both school and sex, the ∼7-year-old TDMo (*F* = 12.759, *p* < 0.001) and ∼11-year-old TDMo (*F* = 12.705, *p* < 0.001) groups maintained significant improvements. However, observed changes from baseline to follow-up remained non-significant in the ∼7-year-old TDMi (*F* = 1.125, *p* = 0.292), ∼7-year-old control (*F* = 0.148, *p* = 0.701), ∼11-year-old TDMi (*F* = 1.395, *p* = 0.242), and ∼11-year-old control (*F* = 0.095, *p* = 0.759) groups.

After adjusting for school effects, significant increases in mean daily school-based step count were observed in the 7-year-old TDMi (*F* *=* 33.357, *p* < 0.001), TDMo (*F* *=* 37.585, *p* *<* 0.001), and control (*F* *=* 6.433, *p* = 0.014) groups, as well as in the ∼11-year-old TDMi (*F* *=* 16.971, *p* < 0.001), TDMo (*F* *=* 7.055, *p* = 0.010), and control (*F* *=* 8.054, *p* = 0.006) groups. Comparably, after adjusting for both school and sex, these improvements remained significant for the 7-year-old TDMi (*F* *=* 40.499, *p* < 0.001), TDMo (*F* *=* 48.777, *p* *<* 0.001), and control (*F* *=* 7.589, *p* = 0.008) groups, along with the ∼11-year-old TDMi (*F* *=* 17.694, *p* < 0.001), TDMo (*F* *=* 9.222, *p* = 0.003), and control (*F* *=* 12.580, *p* < 0.001) groups.

### KIDSCREEN-27

Table 7 presents the mean results for participants in the ∼11-year-old TDMi, TDMo, and control groups who completed the baseline and follow-up KIDSCREEN-27 questionnaire. After adjusting for school effects, TDMo group showed significant improvement in mean KIDSCREEN-27 scores from baseline to follow-up (*F* = 4.561, *p* = 0.036). However, no significant changes were observed among TDMi (*F* = 1.640, *p* = 0.205) or control (*F* = 0.781, *p* = 0.380) groups. Further adjustments for school and sex effects confirmed these findings, with a significant increase in KIDSCREEN-27 scores observed in the TDMo group (*F* = 4.561, *p* = 0.036). Additionally, changes between baseline and follow-up remained non-significant for the TDMi (*F* = 1.643, *p* = 0.204) and control (*F* = 0.787, *p* = 0.378) groups.

**Table 7:** Mean baseline and follow-up KIDSCREEN-27 questionnaire results.

Condition	*N*	Baseline (SD)	Follow-up (SD)
TDMi	35	107.29 (16.22)	111.71 (13.41)^a^
TDMo	34	109.79 (12.65)	115.65 (9.77)^b^
Control	38	111.08 (12.35)	113.05 (9.63)

^a^ Small effect size from baseline.

^b^ Medium effect size from baseline.

### Mean TDMi and TDM enjoyment score: modified PACES questionnaire

A significantly higher mean enjoyment score (*Z* = 2.258*, p* = 0.024, *d* = 0.409) with a small effect size was identified in TDMo participants (*n* = 41, *M* = 4.61) compared to TDMi participants (*n* = 40, *M* = 4.43). After adjusting for the impact of children's sex, no significant difference (*Z* = 0.483, *p* = 0.629, *d* = 0.047) in the mean enjoyment score was observed among boys in TDMi group (*n* = 17, *M* *=* 4.61) and boys in TDMo group (*n* = 22, *M* = 4.63). However, a significant difference with a medium effect size was found (*Z* = 2.432, *p* = 0.015, *d* = 0.605) between girls across both conditions, with girls in TDMo group (*n* = 19, *M* = 4.58) reporting higher mean enjoyment scores than girls in TDMi group (*n* = 23, *M* = 4.30). The mean scores for the six questions added to the modified PACES questionnaire for both TDMi and TDMo groups are presented in Table 8.

**Table 8:** Mean scores to six additional questions for TDMi and TDMo participants*.*

*When participating in TDMi/TDMo* …	TDMi (SD)	*N*	TDMo (SD)	*N*	*Z*	*P*	*D*
I feel it lacks variety	2.85 (1.35)	40	4.15 (1.01)	41	4.283	<0.001	1.102
It allows me to spend quality time with my friends	4.58 (0.75)	40	4.59 (0.87)	41	0.436	0.663	0.012
It is easy to make new friends with children in my class	3.65 (1.08)	40	3.51 (1.27)	41	0.275	0.783	0.119
It helps me to relax and clear my head of negative thoughts	3.83 (0.98)	40	4.17 (1.00)	41	1.764	0.078	0.343
It has a positive impact on my fitness levels	4.18 (1.01)	40	4.71 (0.56)	41	2.662	0.008	0.675
It positively affects my concentration levels when I return to class	3.63 (0.98)	40	3.92 (1.16)	40	1.535	0.125	0.271

### Focus groups

The focus groups further explored the quantitative findings by examining and comparing children and teachers' experiences and perceptions of TDMo and TDMi. Specifically, three key themes emerged that highlighted the similarities and differences between these initiatives; (1) enjoyment, (2) impact, and (3) implementation and sustainability.

#### Enjoyment

Child participants in both TDMi and TDMo shared several common factors that influenced their enjoyment, with the break from the classroom and the opportunity to go outside for fresh air consistently highlighted as key contributors. Although TDMo differs from TDMi in that it can be conducted indoors, it became evident that participating in outdoor activities was generally more enjoyable. Nevertheless, the variety of games implemented during TDMo, helped maintain children's engagement and enjoyment. Additionally, tailoring the delivery of TDMo to meet the needs of all children helped to sustain the engagement of those who were not typically competitive. Conversely, the monotonous nature of the TDMi following the same route each day affected children's enthusiasm for participation. Moreover, the repetitiveness of TDMi often led children to adapt and self-regulate to introduce variety in their movement. However, the use of wearable technology was reported to incentivise children's engagement with TDMi. Illustrative quotes relevant to this theme are presented in Table 9.

**Table 9:** Sub-themes and quotes representing enjoyment in TDMi and TDMo.

Sub-theme of enjoyment	Topic of theme	Quote
Common to both TDMi & TDMo	Fresh air & break from the classroom	“I like that we got to have a break from work and that we got a bit of fresh air every day” (Child, TDMi).
		“If you are having a bad day or doing work and your concentration levels aren't that high, it's nice to get out in the fresh air for the break” (Child, TDMo).
Relevant to TDMo	Outdoor activities are more enjoyable	“Probably less enjoyable inside because we had more boundaries. We weren't allowed to get too excited like we could on the yard where we had loads of space”. (Child, TDMo).
	The adaptability of TDMo effectively enhances engagement among all children	“There is one boy in my class who often cries and hates anything competitive. The second we would step outside, he would cry. But once he realised that TDMo was not competitive – where he didn't have to be first, the best, or win—he really, really enjoyed it. I think for kids like that, this really brought out their enjoyment. It was something that I hadn't seen much of before in some of the quieter, shyer, and less sporty children” (Teacher, TDMo).
	Effect of variety	“I enjoyed it because each week we were able to do a different activity. Sometimes we'd be doing relays, sometimes we'd be doing chasing games like Pacman” (Child, TDMo).
Relevant to TDMi	Effect of monotony	“Sometimes it would get a bit boring when we kept going around the same loop” (Child, TDMi).
	Adjusting participation	“I suppose the lack of variety, they dealt quite well with running around, but I found mine cartwheeling, crawling, doing all sorts and starting to play chase towards the end” (Teacher, TDMi).
	Enhanced engagement through technology	“I have the older class and many of them have wearables and Fitbits. After a few weeks, some children started noticing the step counters on their devices, and the class bought into it a little bit more” (Teacher, TDMi).

#### Impact

Participation in TDMi and TDMo were perceived to result in similar outcomes by focus group participants. Illustrative quotes related to the impact of both initiatives are presented in Table 10. Supporting the results that both TDMi and TDMo positively affected participants' CRF, children consistently reported an improved self-perception of their physical fitness due to their involvement in these initiatives. Teachers attributed positive effects on concentration to participation in these movement breaks. Additionally, TDMi and TDMo were reported to enhance children's social health while also serving as an immediate stress reliever. Moreover, the inclusive nature of both initiatives seemed to encourage and facilitate participation from all children in the class.

**Table 10:** Quotes representing the impact of TDMi and TDMo.

Topic of quote	Quote
Perceived impact on children's physical fitness	“I think I've gotten a lot faster from it and I think I have got a lot fitter” (Child, TDMi).
	“I felt like I could run better and run faster because I was getting fitter” (Child, TDMo).
Effective movement break for enhancing classroom behaviour	“Our concentration levels were definitely better after TDMi. I know the kids loved it. They loved being in the fresh air for the extra 15 min every day. We were able to get a lot more work done afterwards then. It broke up the day nicely for them” (Teacher, TDMi).
	“It definitely helps to reset some students and helps to ground others. Some children need calming down, while others need a boost. We used TDMo as an effective transition tool between lessons or chunks of work” (Teacher, TDMo).
Positive influence on social health and friendship development	“At the start of TDMi, me and [Name of Child Participant] didn't get along together but now we're very good friends” (Child, TDMi).
	“I think they got along with each other much better as the year progressed because they were all playing together in a game, every day” (Teacher, TDMo).
Mental clarity enhancement and stress relief	“It helps to clear your mind” (Child, TDMi)
	“It's good because if you're feeling stressed, you feel better when you come back in” (Child, TDMo).
Inclusiveness of both initiatives supports participation and sustains engagement	“We saw kids who would not usually participate in PE or physical activity but sometimes that was their favourite activity, the stuff they did in TDMo. Whether they were doing it with you, or we were doing it ourselves, they all loved it” (Teacher, TDMo).
	“I have a sporty class for the most part. So, the boys and girls that are sporty loved it, but some of the boys and girls that aren't really into sport got really into it” (Teacher, TDMi).

#### Implementation and sustainability

Time constraints, largely due to the increasing demands of the curriculum, pose a considerable challenge to the sustained implementation of PA initiatives such as TDMi and TDMo. Inclement weather was frequently mentioned as the main barrier to delivery of TDMi, while limited space was reported to hinder children's engagement with the initiative over time. Additionally, it was highlighted that the long-term adoption of the TDMi may be jeopardised without adding an incentive to motivate children. Supporting this, both teachers and children that participated in TDMi consistently suggested introducing variety to counteract the initiative's repetitive nature. However, a teacher's lack of confidence or competence in delivering engaging and novel movement breaks, combined with the absence of external support from a PA specialist, was recognised as a potential threat to the sustainability of TDMo. Furthermore, one teacher identified the pressure to continually deliver new games and activities as a significant challenge to maintaining TDMo implementation. In addition, another teacher suggested delivering TDMo in short blocks throughout the school year, with participatory breaks in between, could positively impact the maintained implementation of the initiative. Explanatory quotes regarding the implementation and sustainability of TDMi and TDMo are provided in Table 11.

**Table 11:** Quotes representing the implementation and sustainability of TDMi and TDMo.

Topic of quote	Quote
Time constraints impact implementation of TDMi & TDMo	“The biggest problem would be the time it requires. We wouldn't be that time-rich to have 25 min every day for it in a real-life setting. Curriculum overload is huge at the moment. That's the hardest challenge that's facing all initiatives, not just this one” (Teacher, TDMo).
	“There have been matches or trips out of school, and if we're out of the classroom for two or three hours, TDMi is the one that's probably pushed out. Teachers are going to move the languages, maths, and literacy to the front of the day if their timetable is already being reduced by outside activities” (Teacher, TDMi).
Inclement weather is a barrier to TDMi delivery	“If it was raining” (Child, TDMi).
	“The weather. That's the only thing I can think of” (Teacher, TDMi).
Effect of introducing an incentive to TDMi	“The repetitiveness of going out every day became a drawback. The introduction of the challenge to complete 100 or 150 laps together was much more enjoyable than the monotonous laps. These challenges were better because they weren't individual, they were team goals. I felt that small differentiation made a big difference. If you stuck to the original TDMi for 15 min and made no changes to it, I don't think it would have been met as enthusiastically by my students” (Teacher, TDMi).
Desire to introduce variety to improve TDMi	“Maybe for 2/3 days do the same thing and then for the next 2/3 days do a different thing” (Child, TDMi)
	“Maybe if there was a big obstacle course for us with some punching bags and some cones that we have to jump between” (Child, TDMi).
	“Just to add some variety to it. Doing 15 min of laps every day does get a bit repetitive after a while. So, maybe we could try adding in different activities each day” (Teacher, TDMi).
Space constraints can hinder children's engagement with TDMi	“We had very limited space, so it was the same routine every day—we couldn't change the routes or anything. After a while, they started messing around and weren't doing what they were supposed to” (Teacher, TDMi).
A teacher's incompetence may affect children's enjoyment in TDMo	Sometimes when you're not here and we are doing something with our teacher, sometimes it would not be as fun because the games might be boring” (Child, TDMo).
Pressure to deliver variety across games is a TDMo implementation challenge	“If I was doing it again myself, trying to think of different things for them to do every day might become a little bit of a challenge after a while” (Teacher, TDMo).
A break from implementation may positively impact the sustainability of PA initiatives like TDMo	“There's so much curriculum-based work that it is hard to commit to an initiative for the long-term. What I often find is the children become a little bit weary with it after a number of weeks so to make it more sustainable, maybe if it was a 6-week block and then there was a break they would be more inclined to get up for it again, because sometimes the novelty of initiatives can wear off if they drag on or they have a long timeframe. So, if we did 6 weeks of TDMo and then we had a 4-week break, they could really look forward to the next block of it” (Teacher, TDMo)

## Discussion

This study represents only the second investigation into the impact of TDMi within primary schools in the Republic of Ireland (25), providing a unique opportunity to contextualise these findings within the broader European research landscape. Previous studies on TDMi have been conducted across various European countries, including Northern Ireland, Scotland, England, Wales, Italy, Holland, and Belgium (17, 20, 22, 23, 26, 52). The present research is the first to evaluate and compare the impact of TDMi on markers of health and enjoyment with a modified version of the initiative that offers a greater variety and choice of PA options. This study found significant improvements in CRF, with large effect sizes observed in TDMo participants and medium effect sizes in TDMi participants from baseline to follow-up. Comparably, extensive research that monitored fidelity to TDMi implementation have previously reported that the initiative positively affects children's CRF (18, 21, 52, 53). These results, suggest that both TDMi and TDMo can positively impact children's CRF, with the findings of this study indicating that TDMo may be more effective in maximising CRF adaptations in children. Additionally, the regular and controlled implementation of TDMi and TDMo in this study aligns with previous research reporting that higher delivery rates of TDMi are associated with greater improvements in children's CRF (52). However, the CR*F* test used in this study (i.e., 550 m Run) differs from those used in other TDMi-related studies (17, 18, 21, 23, 52, 53), limiting direct comparison of CRF findings. Furthermore, consistent with previous research (52), improvements in 550 m performance with a small effect size were also observed in the ∼7-year-old and ∼11-year-old control groups. This suggests that the positive changes observed among the TDMi and TDMo groups may not be solely attributable to their assigned initiatives. Moreover, previous research demonstrates how teachers and children perceive TDMi as having a positive impact on participants' physical fitness (15, 20, 27, 30, 54, 55). The analysis of the questionnaire data in this study indicates that TDMo participants generally perceived greater improvements in their physical fitness compared to TDMi participants. This suggests that TDMo could be more effective in enhancing children's self-efficacy and confidence in their physical abilities than TDMi. As self-efficacy is a well-established predictor of PA behaviour (56), these findings imply that TDMo may promote greater long-term engagement.

This research is the first to investigate the participatory effect of TDMi on FMS competence and, subsequently, is the first to explore and compare its impact on FMS with a modified version. Significant improvements in FMS competence were observed in TDMo groups, with small effect sizes in the ∼7-year-old group and large effect sizes in the ∼11-year-old group. Children do not develop FMS naturally (57), instead, they require structured opportunities to engage in the quality practice of each skill, accompanied by quality instruction and encouragement (58). TDMo presented participants with a variety of games and activities, which required the execution of an extensive range of locomotor and object-control skills. Moreover, brief but targeted instructions and feedback were provided to children who struggled with specific movement patterns pertinent to certain PA games. This focused intervention likely contributed to the significant FMS improvements observed in the TDMo groups. Conversely, TDMi offered participants the opportunity to engage in the meaningful practice of two locomotor skills, running and walking, without the provision of any specific instructions or feedback. This lack of comprehensive FMS practice and guidance may explain the lack of notable change in overall FMS competence within TDMi groups. Furthermore, small effect sizes were observed in both TDMi groups for reductions in locomotor performance. Bolger et al. (12) reported how participation in a school-based PA intervention, focused on providing MVPA opportunities rather than specific FMS development, positively impacted primary school children's locomotor skills as assessed by the TGMD-2. However, the intervention did not lead to significant improvements in object-control skills or overall FMS competence (12). Furthermore, participation in a primary school-based PA intervention specifically designed to develop FMS was associated with significant improvements in mean TGMD-2 scores for locomotor skills, object control skills, and overall FMS competence (12). These findings suggest that the broader variety of PA and movement patterns in TDMo, accompanied with specific instruction and feedback, may provide a more effective approach to the holistic development of FMS in children compared to TDMi. Additionally, the results are consistent with Morgan et al. (59), who reported that the development of FMS is facilitated by PA specialists through the provision of quality school-based practice and guidance for children. Similarly, participation in an 18-week FMS intervention delivered by a qualified specialist resulted in over 85% of child participants (4–5 years) reaching a stage of competency in locomotor, object-control, and stability skills by the post-assessment (60). However, at the 9-week mid-assessment, the percentage of participants classified as competent in these three FMS components ranged from 58%–69% (60), suggesting that the 10-week duration of this study may have been insufficient to capture the long-term effects of TDMi and TDMo on children's FMS. Future research should explore the effect of TDMo participation on children's FMS competence when delivered by teachers without specialised PA expertise. Additionally, studies should track changes in FMS competency levels over a full primary school year or longer.

This study is the first to examine the impact of TDMi and TDMo participation on children's school-based PA behaviour. The results showed that participation in both TDMi and TDMo were associated with significant improvements in children's MVPA, with a large effect size observed in the ∼7-year-old TDMo group. These positive changes align with the findings of Morris et al. (19) who found that children participating in a TDMi session engaged in significantly more MVPA compared to those that experienced a curriculum-based lesson. Comparably, these results are consistent with those of Chesham et al. (17) who reported that participation in TDMi positively impacted children's habitual weekday and weekend MVPA after accounting for age and sex. The findings of this study reinforce the positive association between TDMi and MVPA, while also indicating that both TDMi and TDMo positively influence PA behaviour throughout the school day. Moreover, participation in both TDMi and TDMo was observed to positively impact children's sedentary behaviour across the school day. These findings align with previous research reporting that children spent significantly less time in sedentary positions during a TDMi session compared to those remaining in the classroom for a curriculum-based lesson (19). Similarly, the results support Chesham et al. (17), who reported that children's habitual sedentary behaviour was positively influenced by TDMi participation. However, reductions in school-based sedentary behaviour were only statistically significant among TDMo groups. Although reductions were also noted in TDMi conditions, with a small effect size observed in the ∼11-year-old TDMi group, these changes did not reach statistical significance. The variety of games in TDMo may have encouraged longer engagement from both teachers and children, explaining the greater reduction in sedentary-based behaviour among TDMo participants. Conversely, previous research by Gray and Evans (54) reports that children sometimes engage in TDMi for less than the recommended duration of 15 min, which may limit its impact. Additionally, the option to deliver TDMo indoors when outdoor delivery was restricted may have contributed to the enhanced positive outcome observed for TDMo. Consequently, participation in TDMo may have a higher ceiling effect on reducing children's sedentary behaviour at school than TDMi. Additionally, the follow-up assessment showed significant increases in the mean daily step count for TDMi, TDMo, and control groups. The large effect sizes observed in the ∼7-year-old TDMi and TDMo groups, compared to the small effect size noted in the ∼7-year-old control group, suggests that both initiatives may enhance the PA levels of younger primary school children. However, the medium effect size observed in the ∼11-year-old control group, compared to the medium and small effect sizes in the ∼11-year-old TDMi and TDMo groups, indicates that these initiatives may not result in greater quantities of school-based PA for older primary school children. Furthermore, previous research on school-based PA interventions suggests that the rate and magnitude of improvements in positive PA behaviours tend to diminish over time (61). This highlights the challenge of sustaining long-term improvements in children's PA and reductions in sedentary behaviour at school. The long-term impact of teachers' implementation of TDMi and TDMo on PA behaviours warrants further exploration.

Small and medium effect sizes in HRQoL over time were noted in TDMi and TDMo. However, significant improvements in HRQoL were reported only in TDMo group. This may suggest that the variety and novelty of activities in TDMo have greater potential to positively influence children's health-related wellbeing, compared to the self-paced nature and limited activity choices in TDMi. Nevertheless., the positive changes in reported HRQoL may have been mediated by the seasonal effects of summer and the resulting conditions that facilitated more opportunities for outdoor activity. Keller et al. (62) investigated the impact of weather on mood and found that participants' mood improved when they spent time outside on warm, clear days, supporting the idea that pleasant weather conditions can positively affect wellbeing. Furthermore, the Healthy Choice Programme was developed using teaching practices based on Self-Determination Theory principles to support children's needs. This programme included daily participation in TDMi and weekly hour-long practical sessions that involved discussions about the health benefits of PA (37). The positive change in HRQoL over time observed in TDMi and TDMo groups in this study aligns with Shannon et al. (37), who reported that the Healthy Choice programme enhances children's autonomy, which in turn, positively impacts HRQoL as measured by KIDSCREEN-27. Furthermore, PA is positively associated with a reduced risk of mental health symptoms such as depression, anxiety, tension, and stress, while also enhancing mood, general wellbeing, self-worth, and quality of life (63). This suggests that long-term participation in both TDMi and TDMo could have a positive impact on children's psychosocial wellbeing. However, the improvements observed in HRQoL among TDMi and TDMo participants at this study's follow-up assessment are inconsistent with some prior research focused specifically on TDMi, which did not find a significant effect of participation on measures of children's wellbeing (64, 65). In contrast, Arkesteyn et al. (22) suggested that TDMi may positively impact elements of children's psychological wellbeing, particularly in those with poor mental health. However, these findings should be interpreted cautiously, as the absence of a control group limits the generalisability of results (22). Additionally, a small effect size in favour of TDMo compared to TDMi was found following the analysis of questionnaire data for the initiative's ability to help children relax and clear their heads of negative thoughts. This finding suggests that TDMo may be more effective than TDMi in promoting psychological wellbeing immediately after participation. Future research should further explore the impact of TDMi and TDMo participation on health-related wellbeing while considering factors such children's sex as potential mediators. Moreover, studies should prioritise investigating the long-term effects of these initiatives to better understand their relationship with children's wellbeing.

Results from the modified PACES questionnaire suggest that children's sex influences their enjoyment levels when participating in TDMo and TDMi, with girls reporting significantly higher enjoyment in TDMo compared to TDMi. Additionally, significantly fewer participants indicated that TDMo lacked variety compared to TDMi participants. The variety of PA options that TDMo offers was frequently identified as a key factor influencing children's enjoyment in the focus groups, making it a valuable facilitator for its regular delivery. Conversely, the lack of variety and repetitiveness of TDMi was identified as an implementation barrier, negatively affecting enjoyment and prompting children to self-regulate and adapt their participation. This is consistent with previous research indicating that children may find the monotonous nature of TDMi boring and desire greater variety in their exercise options (20, 25). Furthermore, these findings align with a recently published study that reports increased engagement and enjoyment among children when varied and novel activities are incorporated into the delivery of TDMi (33). Moreover, the inherent focus of TDMi on the locomotor skill of running may not effectively support children's need for autonomy, potentially reducing their engagement and enjoyment (33). Therefore, the inclusion of choice, variety, and new activity options for children in TDMo may promote sustained commitment to the initiative in schools. Conversely, children's lack of involvement and input in the selection of activities, coupled with the repetitive nature of TDMi, may hinder some primary school children's long-term engagement with the initiative. Nevertheless, the introduction of a class-orientated incentive motivated children to participate and effectively counteracted their waning interest in TDMi. This aligns with research highlighting the impact of providing an incentive to sustain children's engagement and motivation in TDMi over time (25, 27). Furthermore, focus group participants perceived both TDMi and TDMo as having a positive effect on children's concentration when they returned to classroom-based teaching and learning. However, these subjective results should be interpreted with caution, as previous research has not found a statistically significant acute effect on children's executive functions following participation in TDMi (19, 20).

The break from classroom-based teaching and the opportunity for outdoor exercise were consistently identified as important factors influencing children's enjoyment across both initiatives. Furthermore, feedback from the questionnaires and focus groups indicated that participants valued the time spent with friends which both TDMi and TDMo were perceived to provide. In addition, the focus group data inferred that rainy conditions had considerably more of a negative impact on implementation of TDMi compared to TDMo. Comparably, inclement weather has consistently been recognised as a barrier to delivery of TDMi (23, 25, 27, 38). Notably, according to Petrigna et al. (66), school-based movement breaks delivered outdoors or indoors, are associated with improvements in children's PA behaviour, motor skills, physical fitness, cognition, and academic attainment. However, the analysis of this study's focus group data revealed that children's enjoyment often decreased when TDMo was conducted indoors, suggesting that prolonged inclement weather could negatively impact the sustained delivery of TDMo. Furthermore, time constraints during a typical school day were acknowledged by teachers as a sizeable threat to the daily delivery of both TDMi and TDMo. Consistent with previous research, time constraints resulting from curriculum demands have been widely referenced as a substantial obstacle to the implementation of TDMi (23–25, 27, 30, 38). Additionally, although TDMo was generally well-received by teachers and children, the pressure on teachers to maintain engagement by regularly introducing new activities may challenge its sustainability. Subsequently, involving children in the decision-making process regarding the activities delivered during a TDMo session may strengthen commitment to the initiative among teachers, staff, and children, and support its long-term-implementation in school settings.

Athletics Ireland coordinates and supports the implementation of TDMi in Ireland. More than one in three Irish primary schools are registered as TDMi participants (16), representing a considerable proportion of schools that may be interested in integrating the TDMo into their policies. Athletics Ireland has already established accessible communication channels with these schools regarding school-based PA. The development of a digital resource that outlines the key implementation principles of TDMo and provides movement break ideas would offer valuable support to schools and teachers considering its adoption. Given that a core implementation principle of TDMo is to keep the delivery of activities simple and avoid unnecessary complexity, teachers are unlikely to require extensive training to effectively implement it with their class group. However, a brief, specialised online workshop facilitated by Athletics Ireland, could ensure teachers are equipped to seamlessly integrate TDMo into their school's habitual patterns and routines. Additionally, establishing a peer-support network would enable schools to share best practices, fostering the sustainability and scalability of the initiative across Irish primary schools. Future research should evaluate and compare the effects of TDMi and TDMo participation over a longer period. It is important to determine whether the findings of this study are consistent over the course of a full primary school year or longer. Moreover, it is imperative to assess teachers' implementation of TDMo without assistance from external coordinators to ascertain the facilitators and barriers associated with the long-term adoption and implementation of the initiative in primary school settings.

## Strengths

The between-subjects design is among this study's strengths as it allows for the comparison of three distinct conditions on participants' health-related metrics. The relatively large sample size (*n* = 268) facilitated the production of a robust data set that enhances the generalisability of the findings. The inclusion of participants' school and sex as random effects helped to improve the accuracy and comparability of findings across conditions. Moreover, the inclusion of both younger and older class groups facilitated a precise analysis of age-specific effects. The implementation of TDMi and TDMo was primarily managed by the researcher and assistant, ensuring any observed effects could be confidently attributed to participation in the respective initiative. Additionally, specific guidelines were provided to the teachers of class groups participating in TDMi and TDMo to ensure consistent delivery and adherence to the implementation principles. The inclusion of a control arm allowed for comparison and assessment of each initiative's effectiveness. Multiple markers of children's health and enjoyment were measured, facilitating a comprehensive overview of each initiative's impact. The Easter break activity likely served to sustain and reinforce the positive health-related behaviours linked to participation in both initiatives.

## Limitations

The convenience sampling method used in this study likely led to the inclusion of schools in TDMi and TDMo conditions that were already motivated to engage in regular school-based PA. Moreover, teachers of participating class groups expressed interest in being involved and were likely keen to ensure the successful implementation of their assigned PA initiative. Consequently, the potential health-related impact of these initiatives on children in schools that do not prioritise a PA lifestyle, lack a culture that values PA, or are taught by teachers who do not fully recognise the importance and benefits of school-based PA, may be underrepresented. The 10-week study duration likely restricted its ability to assess long-term health impacts and sustained engagement with TDMi and TDMo, leaving the long-term effects of participation uncertain. Maturation effects, due to clear baseline imbalances in age among the ∼7-year-old and ∼11-year-old groups, may have influenced the results. As a result, the comparison and generalisability of age-related effects may have been affected. Additionally, the sex sample size for the Mann–Whitney U tests did not meet the calculated power requirements, so findings regarding the mediating effect of sex on enjoyment in TDMi and TDMo should be interpreted cautiously. Missing CRF data from one of the controls schools may have reduced the generalisability of the positive impacts reported for TDMi and TDMo. The impact of TDMi and TDMi on children's HRQoL may be understated because the KIDSCREEN-27 questionnaire was only validated for the oldest participants. Similarly, the enjoyment of the youngest participants in TDMi and TDMo may not be fully represented due to the exclusion of their modified PACES questionnaire data from the analysis.

## Conclusion

Research indicates that TDMi positively impacts children's health related-metrics, namely CRF and PA. However, initiatives like TDMo may offer a more holistic approach to improving children's health. Modifying TDMi to include a greater variety and choice of PA options is likely to boost children's engagement and enjoyment. Consequently, TDMo may offer greater sustainability potential than TDMi. Future research should focus on evaluating the implementation and maintenance of TDMo by teachers throughout the school year, without the assisted delivery of the initiative by trained PA specialists.

## Data Availability

The original contributions presented in the study are included in the article/Supplementary Material, further inquiries can be directed to the corresponding author.
