# Supplementary material for: Comparing the impact of “The Daily Mile™” vs. a modified version on Irish primary school children's engagement and enjoyment in structured physical activity
Source: Front Sports Act Living. 2025 Mar 24;7:1550028. doi: 10.3389/fspor.2025.1550028 (PMC11973259; doi:10.3389/fspor.2025.1550028)
Supplement: Supplementary file 1 [file Table1.docx]

**Supplement 1.** KIDSCREEN-27 questionnaire

**Section B**

1. **Physical Activities and Health**

For each question please tick the most appropriate  for you.

|  |  | **Poor** | **Fair** | **Good** | **Very good** | **Excellent** |
| --- | --- | --- | --- | --- | --- | --- |
| 1 | In general, how would you say your health is? | 1 | 2 | 3 | 4 | 5 |

**Thinking about the last week …**

|  |  | **Not at all** | **Slightly** | **Moderately** | **Very** | **Extremely** |
| --- | --- | --- | --- | --- | --- | --- |
| 2 | Have you felt fit and well? | 1 | 2 | 3 | 4 | 5 |
| 3 | Have you been physically active (e.g., running, biking, climbing) | 1 | 2 | 3 | 4 | 5 |
| 4 | Have you been able to run well? | 1 | 2 | 3 | 4 | 5 |

**Thinking about the last week …**

|  |  | **Never** | **Seldom** | **Quite often** | **Very often** | **Always** |
| --- | --- | --- | --- | --- | --- | --- |
| 5 | Have you felt full of energy? | 1 | 2 | 3 | 4 | 5 |

1. **General Mood and Feelings about Yourself**

For each question please tick the most appropriate  for you.

**Thinking about the last week …**

|  |  | **Not at all** | **Slightly** | **Moderately** | **Very** | **Extremely** |
| --- | --- | --- | --- | --- | --- | --- |
| 1 | Has your life been enjoyable? | 1 | 2 | 3 | 4 | 5 |

**Thinking about the last week …**

|  |  | **Never** | **Seldom** | **Quite often** | **Very often** | **Always** |
| --- | --- | --- | --- | --- | --- | --- |
| 2 | Have you been in a good mood? | 1 | 2 | 3 | 4 | 5 |
| 3 | Have you had fun? | 1 | 2 | 3 | 4 | 5 |
| 4 | Have you felt sad? | 1 | 2 | 3 | 4 | 5 |
| 5 | Have you felt so bad that you didn’t want to do anything? | 1 | 2 | 3 | 4 | 5 |
| 6 | Have you felt lonely? | 1 | 2 | 3 | 4 | 5 |
| 7 | Have you been happy with the way you are? | 1 | 2 | 3 | 4 | 5 |

1. **Family and Free Time**

For each question please tick the most appropriate  for you.

**Thinking about the last week …**

|  |  | **Never** | **Seldom** | **Quite often** | **Very often** | **Always** |
| --- | --- | --- | --- | --- | --- | --- |
| 1 | Have you had enough time for yourself? | 1 | 2 | 3 | 4 | 5 |
| 2 | Have you been able to do the things that you want to do in your free time? | 1 | 2 | 3 | 4 | 5 |
| 3 | Have your parent(s) had enough time for you? | 1 | 2 | 3 | 4 | 5 |
| 4 | Have your parent(s) treated you fairly? | 1 | 2 | 3 | 4 | 5 |
| 5 | Have you been able to talk to your parent(s) when you wanted to? | 1 | 2 | 3 | 4 | 5 |
| 6 | Have you had enough money to do the same things as your friends? | 1 | 2 | 3 | 4 | 5 |
| 7 | Have you had enough money for your expenses? | 1 | 2 | 3 | 4 | 5 |

1. **Friends**

For each question please tick the most appropriate  for you.

**Thinking about the last week …**

|  |  | **Never** | **Seldom** | **Quite often** | **Very often** | **Always** |
| --- | --- | --- | --- | --- | --- | --- |
| 1 | Have you spent time with your friends? | 1 | 2 | 3 | 4 | 5 |
| 2 | Have you had fun with your friends? | 1 | 2 | 3 | 4 | 5 |
| 3 | Have you and your friends helped each other? | 1 | 2 | 3 | 4 | 5 |
| 4 | Have you been able to rely on your friends? | 1 | 2 | 3 | 4 | 5 |

1. **School and Learning**

For each question please tick the most appropriate  for you.

**Thinking about the last week …**

|  |  | **Not at all** | **Slightly** | **Moderately** | **Very** | **Extremely** |
| --- | --- | --- | --- | --- | --- | --- |
| 1 | Have you been happy at school? | 1 | 2 | 3 | 4 | 5 |
| 2 | Have you got on well at school? | 1 | 2 | 3 | 4 | 5 |

**Thinking about the last week …**

|  |  | **Never** | **Seldom** | **Quite often** | **Very often** | **Always** |
| --- | --- | --- | --- | --- | --- | --- |
| 3 | Have you been able to pay attention? | 1 | 2 | 3 | 4 | 5 |
| 4 | Have you got along well with your teachers? | 1 | 2 | 3 | 4 | 5 |
