# Supplementary material for: Comparing the impact of “The Daily Mile™” vs. a modified version on Irish primary school children's engagement and enjoyment in structured physical activity
Source: Front Sports Act Living. 2025 Mar 24;7:1550028. doi: 10.3389/fspor.2025.1550028 (PMC11973259; doi:10.3389/fspor.2025.1550028)
Supplement: Supplementary file 2 [file Table2.docx]

**Supplement 2**. TDMi and TDMo enjoyment questionnaire

School name:

Class group:

Name:

Hello,

Thank you for participating in The Daily Mile/Move over the last number of weeks and for agreeing to complete this questionnaire. By answering the following questions, you will help us to understand the perceptions and feelings that Irish primary school children have when participating in The Daily Mile/Move initiative.

Take your time to read each question carefully and answer it as best as you can. If you are unclear about how to answer any question, please raise your hand and Luke will help you understand how best to answer the question. Please be as factual as possible. It is important that you answer all the questions and that we can see your answers clearly.

Remember we are only interested in your opinions. Your answers will be kept private. You do not have to show your answers to anybody. Also, nobody who knows you will look at your questionnaire once you have finished it.

Thank you!

For each statement please tick the most appropriate  for you.

| **When participating in The Daily Mile / Move ……** | **Disagree a lot** | **Disagree a little** | **Neither agree nor disagree** | **Agree a little** | **Agree a lot** |
| --- | --- | --- | --- | --- | --- |
| 1. I enjoy it | 1 | 2 | 3 | 4 | 5 |
| 2. I feel bored | 1 | 2 | 3 | 4 | 5 |
| 3. I dislike it | 1 | 2 | 3 | 4 | 5 |
| 4. I find it pleasurable | 1 | 2 | 3 | 4 | 5 |
| 5. It is not fun at all | 1 | 2 | 3 | 4 | 5 |
| 6. It gives me energy | 1 | 2 | 3 | 4 | 5 |
| 7. It makes me sad | 1 | 2 | 3 | 4 | 5 |
| 8. It is very pleasant | 1 | 2 | 3 | 4 | 5 |
| 9. My body feels good | 1 | 2 | 3 | 4 | 5 |
| 10. I get something out of it | 1 | 2 | 3 | 4 | 5 |
| 11. It is very exciting | 1 | 2 | 3 | 4 | 5 |
| 12. It frustrates me | 1 | 2 | 3 | 4 | 5 |
| 13. It is not at all interesting | 1 | 2 | 3 | 4 | 5 |
| 14. It gives me a strong feeling of success | 1 | 2 | 3 | 4 | 5 |
| 15. It feels good | 1 | 2 | 3 | 4 | 5 |
| 16. I feel as though I would rather be doing something else | 1 | 2 | 3 | 4 | 5 |
| 17. I feel it lacks variety | 1 | 2 | 3 | 4 | 5 |
| 18. It allows me to spend quality time with my friends | 1 | 2 | 3 | 4 | 5 |
| 19. It is easy to make new friends with children in my class | 1 | 2 | 3 | 4 | 5 |

| **When participating in The Daily Mile / Move ……** | **Disagree a lot** | **Disagree a little** | **Neither agree nor disagree** | **Agree a little** | **Agree a lot** |
| --- | --- | --- | --- | --- | --- |
| 20. It helps me to relax and clear my head of negative thoughts | 1 | 2 | 3 | 4 | 5 |
| 21. It has a positive impact on my fitness levels | 1 | 2 | 3 | 4 | 5 |
| 22. It has a positive effect on my concentration levels when I return to class | 1 | 2 | 3 | 4 | 5 |

**Thank you very much for taking the time to fill out this questionnaire!**
